# The complete mitochondrial genome of *Pheropsophus occipitalis* MacLeay, 1825 (Coleoptera: Carabidae)

**DOI:** 10.1080/23802359.2021.2008825

**Published:** 2022-01-27

**Authors:** Jingru Ke, Dan Guo, Yunzhu Sun, Xufei Pan, Jiayi Ma, Songqing Wu

**Affiliations:** a College of Forestry, Fujian Agriculture and Forestry University, Fuzhou City, China; b Key Laboratory of Integrated Pest Management in Ecological Forests, Fujian Agriculture and Forestry University, Fuzhou City, China

**Keywords:** The mitochondrial genome, *Pheropsophus occipitalis*, phylogenetic analysis

## Abstract

*Pheropsophus occipitalis* MacLeay is a predatory enemy prey heavily on agricultural pests. The length of the complete mitochondrial genome of *P. occipitalis* was 16,800 bp with 20.4% GC content, including 41.2% A, 11.9% C, 8.4% G, 38.5% T. The genome encoded 13 protein-coding genes (PCGs), 22 transfer RNA genes (tRNA), two ribosomal RNA genes (rRNA). Phylogenetic analysis showed that *P. occipitalis* was clustered with *Pheropsophus bimaculatus* and *Pheropsophus sobrinus.* This study provided a scientific basis for the population genetics, phylogeny, and molecular taxonomy of *P. occipitalis*.

*Pheropsophus occipitalis* MacLeay, 1825 (Coleoptera: Carabidae), as the predatory enemy, preys heavily on agricultural pests such as *Mythimna separata* (Lepidoptera: Noctuidae), *Naranga aenescens* (Lepidoptera: Noctuidae), which is conducive to agricultural production and natural balance (Song and Chen 1996). The geographical distribution of *P. occipitalis* includes about 15 countries from Asia, Africa, and Oceania (Venugopal and Thomas 2019). However, the research on this species has focused on its defensive behavior and whether the jet principle can be applied to modern industrial production and military applications (Ji and Ren 2015). To date, the classification of the *Pheropsophus* was based on phenotypic differences, while has received less attention in the genetic evolution (Venugopal and Thomas 2019). In this study, the complete mitochondrial genome of *P. occipitalis* was sequenced and the phylogenetic tree was built to understand the evolutionary relationship of *P. occipitalis*. The results will provide important genetic information for studying the genetic evolution of *P. occipitalis*.

The *P. occipitalis* adults were collected from Minhou, Fuzhou, Fujian province, China (25°51′13′′N, 119°22′29′′E) by the traps with sexual attractants. The specimens are preserved in the Key Laboratory of Integrated Pest Management in Ecological Forests, Fujian Agriculture and Forestry University (URL: https://lxy.fafu.edu.cn, contact person: Jiayi Ma and email: 790167087@qq.com) under the voucher number TN-202102. Total genomic DNA was extracted from an adult using the TruSeq DNA Sample Preparation Kit (Vazyme, China) and purified using QIAquick Gel Extraction Kit (Qiagen, GER). DNA quality and concentration were determined using Nanodrop (Thermo Fisher Scientific, USA). DNA sequencing was performed by Illumina Hiseq 2500 (Illumina, USA). A total of 53,195,560 clean reads were obtained from the 57,498,476 raw reads after filtration. The clean reads were assembled by using MitoZ and metaSPAdes (Nurk et al. 2017). Then the assembly sequence was annotated by the MITOs webserver (Matthias et al. 2013). In addition, tRNA genes were predicted using tRNAscan software (Lowe and Eddy 1997). The complete mitochondrial genome sequence of *P. occipitalis* has been submitted to NCBI GenBank with accession number MW629557.

The results of assembly and annotation showed that the complete mitochondria genome of *P. occipitalis* was 16,800 bp in length. The GC content of the complete genome was 20.4%, including 41.2% A, 11.9% C, 8.4% G, and 38.5% T. And the complete mitochondria genome of *P. occipitalis* encoded 13 protein-coding genes (PCGs), 22 tRNAs, 2 rRNAs. 13 PCGs were 11,202 bp in total, encoding 3733 amino acids, and all PCGs (COI, COII, COIII, ND1, ND2, ND3, ND4, ND4L, ND5, ND6, ATP8, ATP6, and COB) start with a typical ATN codon. Among them, eight PCGs (ATP8, ATP6, COIII, ND2, ND4, ND4L, ND5, and ND6) stop with codon TAA, two PCGs (ND3 and COB) stop with codon TAG, and one PCG (ND1) stop with codon ATC. While the PCGs (COI and COII) stop codon were unusual. The rrnS and rrnL genes were 791 bp and 1328 bp in length, respectively.

To confirm the phylogenetic relationships of *P. occipitalis*, cytochrome oxidase subunit 1 (COI) gene sequences of 13 species related Caraboidea were selected from Genebank BLAST, which the COI gene of *Carabus changeonleei* (GenBank: LC553457.1) was used as an out-group, and the sequence alignment was performed with the MAFFT 7.0 (Katoh and Standley 2013). Then an evolutionary tree was constructed by MEGA 7.0 using the Maximum–Likelihood statistical method and Tamura–Nei model with 1000 bootstrap replicates (Kumar et al. 2016). The phylogenetic tree showed that *P. occipitalis* constituted a monophyletic group with other 5 Carabidae that contains Brachininae, Harplinae, and Carabinae. The monophyletic Carabidae species was assigned to the sister group to the clade of Harplinae in this study. And *P. occipitalis* was clustered together with *Pheropsophus bimaculatus* and *Pheropsophus sobrinus*. Additionally, *P. occipitalis* constituted a paraphyletic group with *Nurus moorei*, *Nurus imperialis*, and *Carabus lafossei* (Figure 1). It is worth noting that *P. occipitalis* is very similar to *P. bimaculatus* but differs by the presence of three spots (anterior and posterior rounded, middle spot narrow connecting the two) on vertex, pronotum with larger spots reaching the lateral margin and narrow median transverse band (Venugopal and Thomas 2019). This study will consolidate the existing knowledge of *P. occipitalis* and provide useful genetic information in phylogenetic and evolutionary studies of *P. occipitalis*.

**Figure 1.**
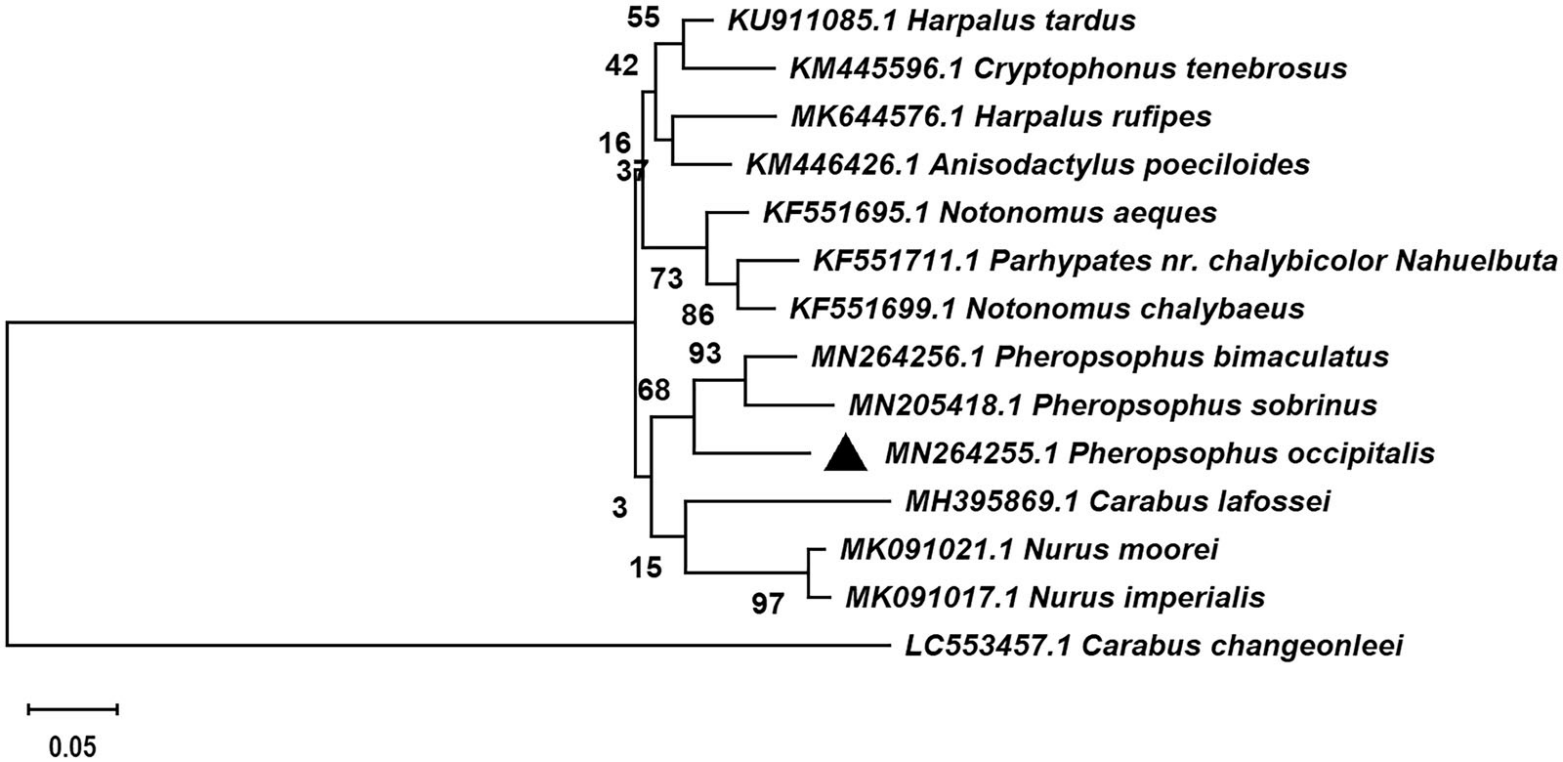
Maximum-likelihood tree of *Pheropsophus occipitalis* MacLeay and related 13 different species insects based on the cytochrome oxidase subunit 1 (*COI*) gene. Bootstrap support values are labeled near the branch.

## Data Availability

The genome sequence data supporting this study’s findings are openly available in GenBank of NCBI at https://www.ncbi.nlm.nih.gov under assessment no. MW629557. The associated BioProject, SRA, and Bio-Sample numbers are PRJNA702873, SRR13743229, and SAMN17981213, respectively.

## References

[CIT0001] Ji LL, Ren GD. 2015. The reserch about bionomics and defensive behaviors of *Pheropsophus occipitalis*. Biotech World. 4(1):2–4.

[CIT0002] Katoh K, Standley DM. 2013. MAFFT multiple sequence alignment software version 7: improvements in performance and usability. Mol Biol Evol. 30(4):772–780. 23329690 10.1093/molbev/mst010 PMC3603318

[CIT0003] Kumar S, Stecher G, Tamura K. 2016. MEGA7: molecular evolutionary genetics analysis version 7.0 for Bigger Datasets. Mol Biol Evol. 33(7):1870–1874. 27004904 10.1093/molbev/msw054 PMC8210823

[CIT0004] Lowe TM, Eddy SR. 1997. tRNAscan-SE: a program for improved detection of transfer RNA genes in genomic sequence. Nucleic Acids Res. 25(5):955–964. 9023104 10.1093/nar/25.5.955 PMC146525

[CIT0005] Matthias B, Alexander D, Frank J, Fabian E, Catherine F, Guido F, Joern P, Martin M, Peter FS. 2013. MITOS: improved de novo metazoan mitochondrial genome annotation. Mol Phylogenet Evol. 69(2):313–319. 22982435 10.1016/j.ympev.2012.08.023

[CIT0006] Nurk S, Meleshko D, Korobeynikov A, Pevzner PA. 2017. metaSPAdes: a new versatile metagenomic assembler. Genome Res. 27(5):824–834. 28298430 10.1101/gr.213959.116 PMC5411777

[CIT0007] Song HY, Chen CM. 1996. Catalogue of natural enemies of rice pests in Hunan Province I. J Hunan Agric Univ. 22(4):39–52.

[CIT0008] Venugopal AS, Thomas SK. 2019. Bombardier beetles of the genus *Pheropsophus Solier* 1833 (Carabidae: Brachininae: Brachinini) from Indian subcontinent. Zootaxa. 4608(1):65. 10.11646/zootaxa.4608.1.3 31717160

